# How Predictability of Feeding Patches Affects Home Range and Foraging Habitat Selection in Avian Social Scavengers?

**DOI:** 10.1371/journal.pone.0053077

**Published:** 2013-01-03

**Authors:** Sophie Monsarrat, Simon Benhamou, François Sarrazin, Carmen Bessa-Gomes, Willem Bouten, Olivier Duriez

**Affiliations:** 1 Centre d’Ecologie Fonctionnelle et Evolutive, UMR 5175 CNRS, Université Montpellier 2, Montpellier, France; 2 Conservation des Espèces, Restauration et Suivi des Populations, UMR 7204 MNHN-CNRS-UPMC, Paris, France; 3 Ecologie, Systématique et Evolution, UMR 8079 CNRS, Université Paris Sud, Orsay, France; 4 Computational Geo-Ecology, Institute for Biodiversity and Ecosystem Dynamics, University of Amsterdam, Amsterdam, The Netherlands; Columbia University, United States of America

## Abstract

Feeding stations are commonly used to sustain conservation programs of scavengers but their impact on behaviour is still debated. They increase the temporal and spatial predictability of food resources while scavengers have supposedly evolved to search for unpredictable resources. In the Grands Causses (France), a reintroduced population of Griffon vultures *Gyps fulvus* can find carcasses at three types of sites: 1. “light feeding stations”, where farmers can drop carcasses at their farm (spatially predictable), 2. “heavy feeding stations”, where carcasses from nearby farms are concentrated (spatially and temporally predictable) and 3. open grasslands, where resources are randomly distributed (unpredictable). The impact of feeding stations on vulture’s foraging behaviour was investigated using 28 GPS-tracked vultures. The average home range size was maximal in spring (1272±752 km^2^) and minimal in winter (473±237 km^2^) and was highly variable among individuals. Analyses of home range characteristics and feeding habitat selection via compositional analysis showed that feeding stations were always preferred compared to the rest of the habitat where vultures can find unpredictable resources. Feeding stations were particularly used when resources were scarce (summer) or when flight conditions were poor (winter), limiting long-ranging movements. However, when flight conditions were optimal, home ranges also encompassed large areas of grassland where vultures could find unpredictable resources, suggesting that vultures did not lose their natural ability to forage on unpredictable resources, even when feeding stations were available. However during seasons when food abundance and flight conditions were not limited, vultures seemed to favour light over heavy feeding stations, probably because of the reduced intraspecific competition and a pattern closer to the natural dispersion of resources in the landscape. Light feeding stations are interesting tools for managing food resources, but don’t prevent vultures to feed at other places with possibly high risk of intoxication (poison).

## Introduction

Human intervention on wildlife through conservation programs raises many questions about its impacts on populations and individual behaviour. Behaviour has an important affect on the viability of a population since the strategies chosen by individuals influence demographic parameters such as dispersal, survival and reproduction [1]. To increase population viability of threatened species, wildlife managers often use food resource management through the use of supplementary feeding [2], [3] particularly in the case of raptor conservation programs [4], [5], [6]. For the conservation of obligate scavengers like vultures, resources are usually managed through the use of feeding stations, where carcasses are intentionally left for vultures to provide them with food [7]. In India, feeding stations are supposed to provide a safe food source after millions of vultures were decimated by the contamination of carrion by a veterinary drug in the 1990s [8], [9]. This conservation measure is also used to provide carcasses in areas with insufficient food [10], to increase survival [11], to facilitate the recolonization of abandoned breeding sites [7] and to support reintroduction programs [12], [13].

This gathering of food inevitably results in an increase of the spatial and temporal predictability of food resources but the nature of this impact on birds’ foraging behaviour and population dynamics is still debated [14], [15]. Social vultures which naturally search for largely unpredictable food resources (carcasses) may face a risk of ecological trap when managed with feeding stations because their behavioural responses inherited from their evolutionary history may be inappropriate in a new context of predictable food resource [16], [17]. In solitary and territorial Bearded vultures *Gypaetus barbatus for example*, young individuals tend to concentrate around feeding stations and this prevents the colonization of new sites, where food may be harder to find [6]. In this species, supplementary feeding did not improve the breeding success and even resulted in a lower productivity by changes in breeding strategies [18], [19]. However, the aggregation around carrions being an expected element in the life-history of social species of Old world (e.g. *Gyps spp* in the Eurasia and Africa) and New world (e.g. *Coragyps atratus* and *Cathartes spp*) vultures, the impact described for solitary species like the bearded vulture may not apply. Yet impacts of feeding stations like habituation have been suspected as well on social vultures but have never been properly demonstrated [20].

The population of Eurasian griffon vultures *Gyps fulvus* reintroduced in 1980 in the Grands Causses in France [13] provides an interesting opportunity for understanding the impact of feeding stations on social vultures. As part of the reintroduction program and for sanitary legislation reasons, managers established two types of feeding stations. First, managers developed “Heavy Feeding Stations” (HFS), equivalent to vulture restaurants used in Africa [21], where carcasses from nearby farms are collected and concentrated at a few sites. Second, managers encouraged farmers to install “Light Feeding Stations” (LFS) where they can leave the few carcasses from their own herd at a dedicated place in their farm. LFS network helped diluting the concentration of carcasses and enlarging the area containing resources. Meanwhile, vultures can also benefit from persisting traditional (although illegal) practices to leave carcasses in the fields at free disposal for scavengers. Regarding food predictability, HFS can be considered as highly predictable in time and space, while LFS are only predictable in space and illegal deposits remain unpredictable. Moreover, variation throughout the year in mortality in domestic sheep, i.e. the amount of resource available for vultures, provided a complementary test of the impact of temporal food predictability on vulture behaviour. Due to rearing practices, carcasses were more numerous in winter and spring, at the start of the vulture breeding season, than in summer and autumn when chicks are fledging [22], [23]. Conditions for flight also change throughout the year, relative to insulation that creates thermal convections. In Mediterranean climates, days are longer and sunnier in summer than in winter [24]. Hence, thermals being stronger and more numerous in summer than in winter, vultures can fly longer distances in summer. To sum up, the availability of food resources in the Grands Causses provided a quasi-experimental framework to tackle the question of impact of feeding sites on the foraging behavior of griffon vulture: the network of HFS and LFS gave insight into spatial predictability of food resources while seasonality along with flight conditions, affected the availability, hence temporal predictability, of food resources.

Vulture foraging behaviour was explored through intensive GPS tracking. Individual home ranges were first estimated and compared between seasons, ages and sexes. Then, habitat selection analyses were performed to detect whether vultures preferred to forage over habitats with predictable or unpredictable resources, and whether this preference varied between seasons (with availability and flight conditions). We hypothesized that the use of the most predictable resources should increase when the overall amount of resources available decreases. In the Causses, this effect is likely to appear in summer, when the carcasses are scarce. In winter, when carcasses are abundant, albeit compensated by limited flight abilities, we expected a reduction in the use of the most predictable resources, as a mean to reduce the intense intraspecific competition [22], [25], [26]. Foraging behaviour was likely to differ between age classes for two reasons. First immature vultures were dominated by adults at feeding events [22]. Second, being non-breeders, immatures were also less restrained in their foraging behaviour (at least during the breeding season) because they do not need to return to a nest every evening and they have no chick to feed (i.e. lower energy requirement). Thus, immature birds were expected to display larger home ranges than adults, as previously described with radiotracking data [27]. Finally, as the dimorphism between males and females is almost non-existent and as both sexes are known to share parental duties equally [28], we did not expect to find differences in foraging behaviour between sexes, in agreement with previous studies on other vulture species [29], [30].

## Materials and Methods

### Ethics Statement

The study was not specifically approved by an ethical committee as a permit for equipping vultures with loggers was provided as part of the licence of Olivier Duriez and Philippe Lécuyer from the Centre for Bird Population Studies (CRBPO) of the Natural History Museum (MNHN, Paris): according to the French law of 22 September 2008, the CRBPO has the delegation by the Ministry of Ecology, Energy, Sustainable Development and Land Settlement for allowing the owners of a general bird ringing licence to capture and handle birds from protected species, and mark them (with rings or any other device like GPS units). Birds were also handled under the permit of the Ligue pour la Protection des Oiseaux (national certificate to maintain birds (“Certificat de capacité”) number 12–251 delivered to Philippe Lécuyer (authorized ringer) on 5 July 2005). All care was taken to reduce any potential disconfort to the birds: to reduce stress of birds, the head was covered by a tissue and handling time was reduced to minimum (<20 min). Logger mass was <1% of bird body mass, ie<the 3% generally recommended for flying birds. Logger harnesses were designed to fall off after a few years to prevent these long-lived birds from carrying the logger for the rest of their lives.

### Study Area and Vulture Population

**Figure 1:**
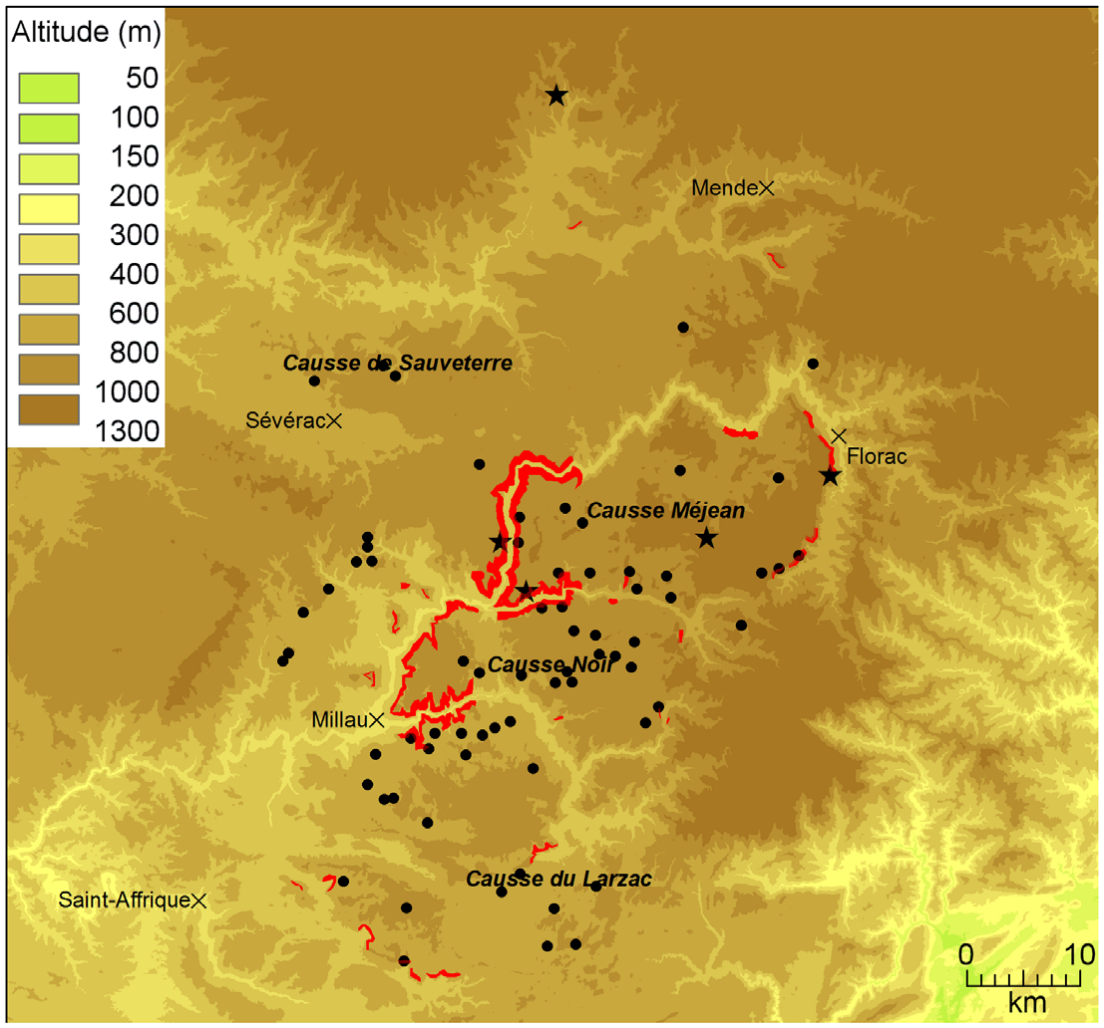
Study area map. Breeding colonies are represented in red, light feeding stations (LFS) as black dots, heavy feeding stations (HFS) as black stars and main cities as black crosses. The name of the major topographical features such as limestone plateaus (Causses) and mountain ranges are written in bold.

The Grands Causses (southern France) consist of four limestone plateaus (Causses Sauveterre, Méjean, Noir, and Larzac) separated by deep canyons (Tarn, Jonte and Dourbie canyons; Fig. 1). Farming in the Causses mostly involves extensive sheep rearing and grazing in grassland semi-steppic landscape. In 2011, carcasses of livestock could be legally deposited for vultures at five HFS (depending on season, between 1 and 10 carrions daily) and at 69 LFS (on average 1 carcass per month). Some additional resources might occur outside these legal sites, giving vultures an opportunity to feed on randomly distributed resources. However no information about carcasses dropped outside feeding stations was available.

After the successful reintroduction in the 1980s by the Parc National des Cévennes (PNC) and the Ligue pour la Protection des Oiseaux (Birdlife France) [13], more than 300 pairs of griffon vultures were breeding in the area in 2010 (LPO, unpublished data). More than half of the individuals were marked as chicks on the nest, ensuring a high proportion of birds with known age [31]. Griffon vultures lay a single egg between late December and February and incubate for 2 months [28]. The chick is brooded and fed by both parents between March and July. Fledging occurs from mid-July to the end of August. Postfledgling emancipation is progressive and juveniles are fed at nest until October-November, before starting a period of immature erratism. In this study we considered two age classes: immatures (≤5 years old; because data were available from only 1 juvenile, it was pooled with immatures), and adults (≥6 years old). Sex of all but one individual was determined using molecular techniques based on CHD genes using feather samples collected when ringing chicks [32], [33].

### GPS Tracking

Forty-two vultures of known origin (natal nest) and age (determined with individual rings) were equipped with GPS units. For this purpose, they were captured in summer-autumn 2010 (using an aviary with a remote-controlled door) close to the most visited HFS (Cassagne). We focused here on the movement data collected between June 2010 and September 2011. Individuals tracked for less than 50 days in a single season (table 1) were removed from analysis.

**Table 1:** Number of griffon vultures of each age class and sex tracked by GPS in each season.

Age	Sex	Summer 2010	Autumn 2010	Winter 2011	Spring 2011	Summer 2011
Adult	F	5	4	3	3	2
	M	15	11	8	7	6
	Unknown	1	1	1	1	1
Immatures	F	4	4	4	4	4
	M	2	3	2	1	2
Total		27	23	18	16	15

(Immatures: 0–5 years, adults: >6 years). As the study started in summer 2010, the sample size decreased with the progressive battery shortage or physical loss of GPS units.

The GPS units weighed about 60 g (<0.8% of the body mass). They were attached with a highly resistant, relatively elastic and non-abrasive Téflon harness, secured by the leg-loop method [34]. A toric seal was used as a weak link to release the bird from the harness after about one year. Two types of units were used: SAFT battery-powered GiPSy2 (Technosmart, Italy; http://www.technosmart.eu) and solar-powered UvA-BiTS (Birdtracking system, Amsterdam University, Netherlands; http://www.uva-bits.nl). GiPSy2 units were programmed to get a fix every 5 minutes during the day for a life time of about 6 to 9 months. UvA-BiTS units were programmed to get a fix every 5 to 10 minutes during the day in summer, but limited to only every 15 to 60 minutes in winter, due to lower insulation and shorter days. Thanks to their eight solar cells, they could work as long as the birds carried them. During the night (when the birds were at rest) the fix rate was reduced to a location every hour for both types of unit. Data downloading (and possibly changes in programming) for GiPSy2 units was done manually with a Bluetooth connection operating within 150 meters (performed from a hide close to the Cassagne HFS). In turn, UvA-BiTS data transfer relied on automatic communication scheme between each unit and a network of antennas connected to a base station via the wireless Zigbee technology. Information transmission was possible at distances larger than 10 km, but required birds to be relatively immobile for a long period of time (i.e. feeding or resting in cliffs). The network of five antennas covered two HFS and the main breeding colonies of the Tarn and Jonte canyons. Both types of GPS units provided locations with an accuracy of a few meters (the mean distance between successive locations acquired at 5 min interval with a GPS unit set at a fixed location was always less than 10 m). They were assigned at random to the different age and sex classes.

Unlikely GPS locations were filtered out. First we discarded fixes computed through the use of only 3 satellites and characterized by a dilution of precision larger than 5 or computed with at least 4 satellites and characterized by a dilution of precision larger than 15 [35]. Fixes involving a mean movement speed (between two successive locations) larger than 110 km/h were also discarded. As the time series of locations appeared to be stationary during the whole study period, they could not be segmented on a biologically relevant movement-based basis (e.g. see [36]). To compare movement parameters and home ranges at different periods of the year, we arbitrarily split each time series according to the solstice and equinox dates, as this procedure fitted quite well with the breeding cycle of vultures (incubation between mid-December and mid-March, chick rearing until July, chick fledging in July-August, chick emancipation from September to December).

### Home Ranges Estimation

For each individual and each season, we computed the activity utilization distribution (UD) through Kernel Density Estimation (KDE). To do so, we excluded all resting locations, i.e. diurnal locations within 100 m of each other and all nocturnal locations. Home range and core areas were respectively defined as the areas encompassed within 95% and 50% UD isopleths. For GPS locations acquired at 5–10 min interval during the day, and thereby highly autocorrelated, we used a movement-based KDE [37], [38]. In this case, the smoothing parameter was computed from the diffusion coefficient of movement, estimated to be about 1,3.10^5^ m^2^/min (see [38] for details). By explicitly incorporating local movement information supplied by serial correlation, this method has the advantage to require a smaller smoothing factor than the classical (location-based) KDE, and thus to estimate the UD with a finer spatial resolution. For locations acquired at larger intervals during the day (from 15 to 60 minutes, as occurred with solar-powered GPS in winter), we used the classical KDE [39]. The choice of the smoothing parameter in location-based KDE is ever a hard-to-solve issue [40], [41]. We took advantage of the movement-based UDs to estimate a reliable location-based smoothing parameter. For this purpose, we subsampled locations acquired at 5 min interval to simulate sets of locations acquired at lower frequencies, from which we computed location-based UDs using various values of the smoothing parameter. It turned out that the value leading to location-based UDs closest to the initial movement-based UDs was equal to 0.5 σ *n* ^−1/6^, where σ is the standard deviation of the locations and *n* is the sample size (i.e. half the optimal value expected for a bivariate circular normal UD).

For additional analyses, we considered the following four basic variables: 1. home range area (within 95% UD isopleths); 2. core areas (within 50% UD isopleths), 3. number of feeding stations (HFS and LFS pooled) encompassed in the home range, and 4. mean distance covered per day. From these 4 variables, we derived two additional variables: 5. the density of feeding stations within the home range area (in feeding stations per km^2^); and 6. the “flatness” of the UD, calculated as the ratio core area/home range area (where a value close to 0.53 will indicate a uniform space use, whereas smaller values will correspond to more uneven space use: e.g. for monomodal UDs, a value of 0.23 is expected for a bivariate normal distribution, and of 0.125 for a bivariate exponential distribution). The existence of potential correlations between these variables was tested with the Spearman rank test in order to keep only non-correlated variables for the subsequent statistical analyses. For the 14 individuals tracked during both summer 2010 and 2011, tests of comparison between both summers didn’t reveal any significant difference for the 6 variables considered (Wilcoxon signed-rank test, p>0.05). We thus considered an average of the two summers in the GLMM analysis (see below).

### Foraging Habitat Selection Analysis

Five types of “habitats” were considered in this study: (1) the vulture colonies, corresponding to the canyons (cliffs and the valleys between them) where vultures breed or rest at night but did not forage, (2) the five HFS, each surrounded by a 1-km radius buffer, corresponding to the distance within which a flying vulture is assumed to visually identify a carcass [42], (3) the 69 LFS, also surrounded by a 1-km radius buffer, (4) “open habitat”, i.e. grassland and fields, where vultures can naturally find carcasses, and (5) “unsuitable (foraging) habitat”, i.e. forests, urban areas and water zones, where vultures are not expected to find any food resources as they use only their sight to localize carcasses. These habitat types were identified using SOeS/CORINE Land Cover 2006 (http://sd1878-2.sivit.org/). We analyzed foraging habitat selection by using compositional analysis [43]. This method makes it possible to determine whether a given habitat type is significantly over- or under-used with respect to its random use (expected when the intensity of use for any given quadrat does not depend on the habitat type to which it belongs). In studies focusing on habitat selection within a home range, the UD provides a reliable estimate of the actual use of any part of a home range [44]. However, the random use of a habitat type is classically assumed to correspond to what is called its “availability”, computed as its relative occurrence in the environment. This approach implicitly involves uniformly distributed random use (expected when the intensity of use for any given quadrat is independent of both the habitat type to which this quadrat belongs and of its location in space), which is inadequate for central place foragers such as vultures that generally start their foraging trip from the breeding colony. Indeed, a putative central place forager showing no particular preference for any type of habitat will visit places far from the central place less often than places closer to it. We therefore performed a compositional analysis by comparing the actual use of each habitat type by a given vulture at a given season (as revealed by the UD) with a random use reference based on a circular bivariate exponential distribution, which appears to be the most appropriate random model for this purpose [45], [46], centered on HFS Cassagne (approximately located at the geographical barycentre of the colony), truncated at the 0.95 isopleth. The probability density at any location (*x,y*) located at distance *D* from Cassagne is therefore given by *f*(*x,y*) = 3exp(-√3*D*/σ*_i_*)/(2πσ*_i_* ^2^) for *D<D_max_*(*i*) and *f*(*x,y*) = 0 for *D*>*D_max_*(*i*), where σ*_i_* is the standard deviation, adjusted for each individual *i* to a value such that the radius of the exponential UD corresponds to the distance *D_max_*(*i*) between Cassagne and the furthest location lying on the 0.95 isopleth of the actual UD of individual *i*: σ*_i_* = *D_max_*(*i*)/2.74. Because we were interested in the habitat selected during foraging flight, breeding colonies were excluded from foraging habitat selection analyses, which therefore focused on the following four habitat types: HFS, LFS, open habitat, and unsuitable habitat. Actual habitat use and expected random use (no habitat preference) by any given individual were then computed as the actual UD-weighted and the exponential UD-weighted, respectively, proportions of each habitat type. In the compositional analysis, the choice was made to perform a unilateral test when comparing the use of open versus closed habitat (with a predicted preference for the open habitat) and bilateral tests for every other comparison.

### Other Analyses

To evaluate the effect of season and individual variation on home range characteristics, we used Generalized Linear Mixed effect Models (GLMMs), through the *lmer* function from R (lme4 package) [47], [48]. To correct for pseudoreplication of the same individuals tracked over several seasons, we performed GLMM in two steps with the individual included as a random effect. Step 1: a first analysis was performed considering the variables seasons, age, and age*season as fixed factors. Step 2: on a dataset reduced to adults, a second set of models were built to consider the effect of the sex of individuals, by considering the variables season, sex and sex*season. This procedure enabled to limit the confounding effect between the age and the sex of the individuals, since assigning a sex for the immatures was not relevant in our analyses. We then used the procedure described by Bolker [49]. GLMM were fitted with the Restricted Maximum Likelihood. For the counting variable “number of feeding stations”, GLMMs were used with Laplace approximation. Model selection was made using a backward stepwise method based on the Akaike Information Criterion, as recommended by Bolker [49]. The models selected were then tested for normality and homoscedasticity of residuals using a graphical procedure. Note that, for GLMM, the *lmer* function provides estimates of the fixed-effects parameters, standard errors for these parameters and a *t*-ratio but no *p*-values, as the degree of freedom cannot be known [50]. Consequently, we reported the *p*-values computed in the *lme* function (package nlme). In order to test for differences among the different levels of each parameter, Tukey post-hoc tests were used, with the *glht* (multcomp) function from R. The statistical significance was set at p = 0.05. The values reported in the Results section correspond to mean ± standard deviation.

## Results

Twenty-eight vultures, for which at least 50 consecutive days of monitoring were available, were considered in the analyses (Table 1). The number of locations recorded per individual was 28 628±18 004 for all the study period. However for birds tracked with UvA-BiTS GPS units, the average number of locations was larger in summer (11 725±11 171) than in winter (965±1029).

**Figure 2:**
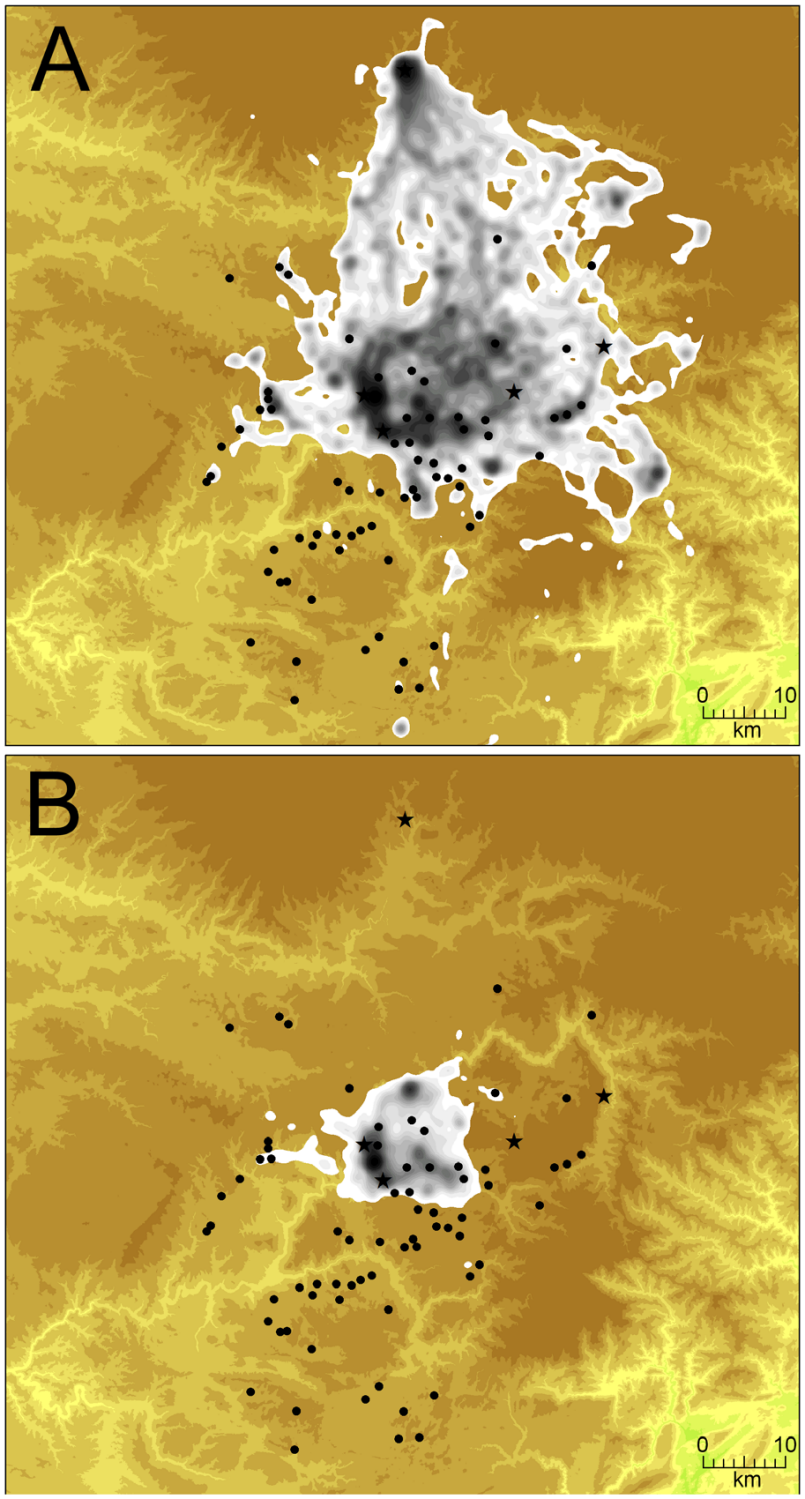
Examples of home ranges of two griffon vultures in summer. (A) a 30 year-old female (HR area = 1 735 km^2^) and (B) a 25 year-old male (HR area = 254 km^2^), illustrating their high individual variability. Areas encompassing higher values of Utilization Distribution (UD) are darker. Light feeding stations (LFS) are represented by black dots and heavy feeding stations (HFS) by black stars. The scale and altimetry are the same as in Fig. 1.

The home range area, with all individuals and all seasons pooled, was 962±623 km^2^, and the core area was 109±80 km^2^. A general gross home range estimated with Minimum Convex Polygon (MCP) method pooling all individual covered >10000 km^2^ and encompassed all the feeding sites. Home range size was maximal in spring and minimal in winter (1272±752 km^2^ and 473±237, respectively). There existed large variations between individuals both in terms of size (range: 256–3143 km^2^ in spring, 44–888 km^2^ in winter) and shape and position around the colony (Fig. 2). Home ranges were generally not circular and centred on the colony: their shapes usually testified for a privileged movement direction.

**Figure 3:**
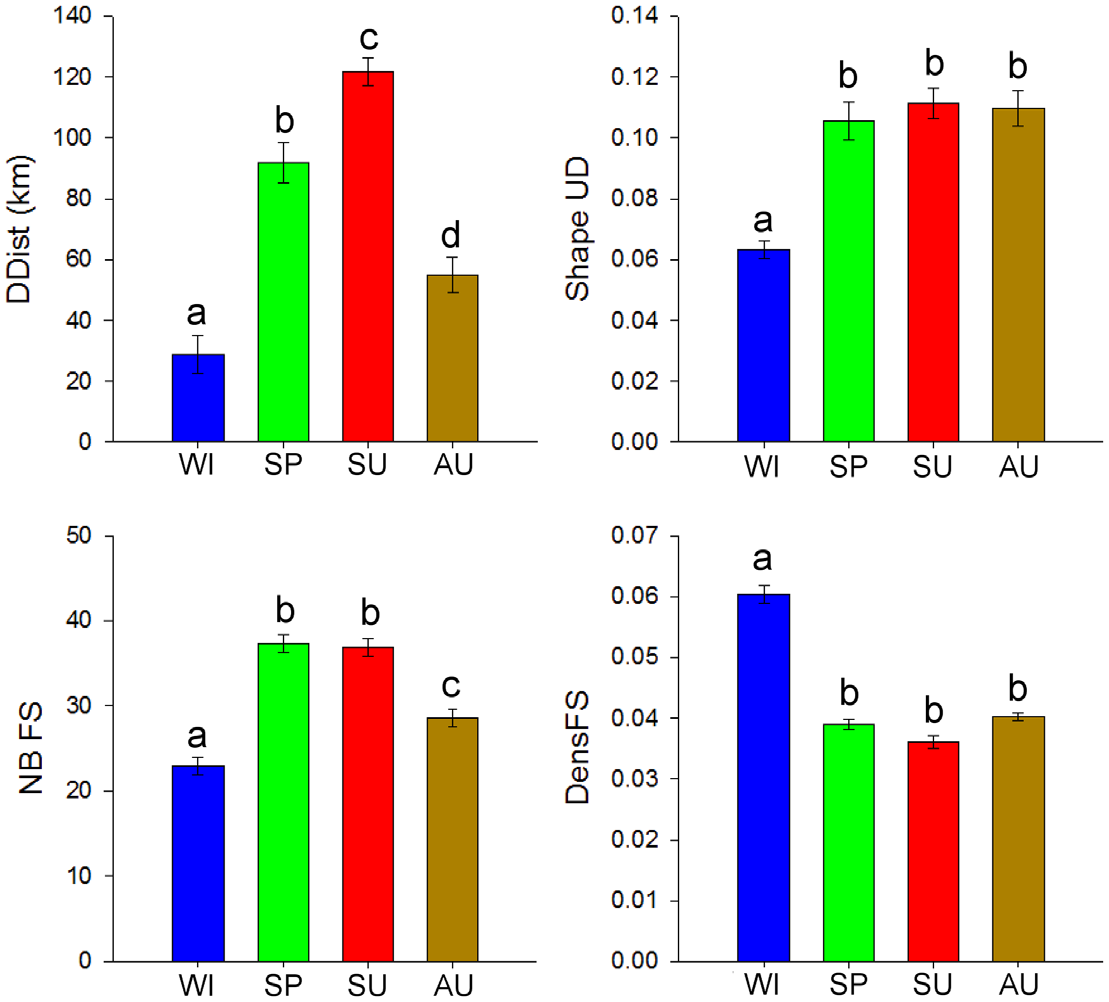
Seasonal differences between the four analyzed variables. Seasonal differences in mean distance covered per day (DDist, in km), flatness of the UD (ShapeUD, corresponding to the ratio core area/home range area), number of feeding stations whithin the home range (NbFS) and density of feeding stations within home range (DensFS, in NbFS per km^2^). The values correspond to estimates (mean±SEM) provided by a GLMM (see Table 2). Letters indicate groups determined by post-hoc tests. WI = winter, SP = spring, SU = summer, AU = autumn.

Among the 6 studied variables, several were significantly correlated (p<0.05): home range area was strongly correlated with core area (r_s_ = 0.84), the number of feeding stations encompassed (r_s_ = 0.79) and the density of feeding stations (r_s_ = −0.85). The core area was also significantly correlated with this density (r_s_ = −0.75). Home range and core areas were therefore excluded from the GLMM analysis, which tested the influence of age, sex and season on the four remaining variables, assumed to be statistically independent: the flatness of the UD, the mean distance covered per day, the number and the density of feeding stations within the home range. All four variables varied significantly between seasons but not regarding age or sex (Table 2). We thus only presented graphs exhibiting seasonal differences provided by the models, with all other individual categories pooled (Fig. 3). The flatness of the UD, which is an index of the concentration of the activity within the home range, was similar in summer, autumn and spring (ca. 0.11), but was much smaller in winter (0.06±0.003). The mean distance covered in a day by vultures was 77.0±40.1 km. It was significantly higher in summer (121.9±4.4 km) than in spring (91.7±6.6 km), autumn (55.0±5.8 km) and winter (28.6±6.4 km). On average, the home ranges included 35.9±13.4 feeding stations. More feeding stations were encompassed in home ranges in summer and spring (36.9±1.1 and 37.3±1.1 respectively) than in autumn (28.6±1.1) and winter (22.9±1.1). Finally, the density of feeding stations within home range was significantly higher in winter (0.060±0.002) than in the other seasons (0.038±0.001). As the global number of feeding station is constant throughout the year, this result indicates that home range was more concentrated around feeding stations in winter.

**Table 2:** Influence of season on four variables, resulting from GLMM analysis.

	Effect	Df	F	P
Mean distance covered per day	Season	3	111.887	<0.0001 ***
Shape of Utilization Distribution	Season	3	9.388	<0.0001 ***
Number of feeding stations	Season	3	16.174	<0.0001 ***
Density of feeding stations	Season	3	30.775	<0.0001 ***

The p-values are those given by the lme (lme4) function. (See Methods).

Foraging habitat selection analysis was performed separately for each season and for adult and immature birds. The compositional analysis (Table 3) detected a significant preference of adults for the most predictable resources, i.e. feeding stations (both LFS and HFS), compared to the other habitats all over the year. In contrast, immature birds did not show any significant preference between the different types of resources in the spring. A significant preference for LFS over HFS in spring and summer was detected for adults, whereas such preference was not identified in immatures. Open (favorable) habitats were also significantly preferred over unsuitable (forested or urbanized) habitats in most seasons (for adults in summer, autumn and winter and for immatures in summer).

**Table 3:** Seasonal habitat selection by adult and immature griffon vultures, obtained by compositional analysis.

Season	Age	n	Wilk’s λ	P	Habitat ranking
Winter	Ad	12	0.020	<0.001	LFS>HFS>>closed habitat>>open habitat
	Imm	6	0.027	<0.05	LFS>HFS>>closed habitat>>open habitat
Spring	Ad	11	0.072	<0.001	LFS>>HFS>>open habitat>closed habitat
	Imm	5	0.005	<0.01	LFS>HFS>open habitat>closed habitat
Summer	Ad	20	0.064	<0.001	LFS>>HFS>>open habitat>>closed habitat
	Imm	7	0.011	<0.001	LFS>HFS>>open habitat>>closed habitat
Autumn	Ad	16	0.063	<0.001	HFS>LFS>> open habitat>>closed habitat
	Imm	7	0.015	<0.001	LFS>HFS>> open habitat>closed habitat

Habitat types were ranked from the most preferred to the least preferred. A significant preference between two habitats was indicated by “>>” while a non significant difference was indicated by “>”. P values were obtained by random permutations. HFS: heavy feeding station, LFS: light feeding station.

## Discussion

Sanitary legislation on carrion management generated a quasi-experimental landscape to assess the impact of food management on the spatial ecology of obligate scavengers, particularly their foraging activity. The analysis of home ranges, daily flight distances and habitat selection, obtained using high resolution GPS devices, demonstrated that vulture foraging strategies could be affected by changes in spatial resource predictability (presence of feeding sites) and temporal resource availability (seasonality of carcasses) related to the management of supplementary feeding.

### Home Ranges

The area foraged by vultures largely encompassed the Grands Causses and, in spring and summer, extended beyond this area towards the Massif Central in the North or the Mediterranean coast in the south. However, when individuals were considered separately, home ranges were not uniform and concentric around colonies (as it could be expected for central-place foragers) but were usually oriented toward one or several specific areas, indicating different foraging areas chosen by individuals. Similar oriented foraging movements were recorded in other typical central place foragers such as albatrosses [51], [52] or penguins [53]. We recorded examples of two birds nesting at short distance in the Jonte Canyon but one individual had a home range centered on the north of the canyon (ie Causse Méjean and Sauveterre) while the other bird was mostly foraging south of the Canyon (Causses Noir and Larzac). This suggests that vultures do not forage completely at random but favour some specific areas.

Only a few studies have estimated home ranges of Eurasian griffon vultures, limiting comparisons. We found average home range areas of ca. 1000 km^2^ and core areas of ca. 100 km^2^. Only one recent study of GPS tracked griffon vultures in Spain described a median home range of 4078 km^2^ and core area of 489 km^2^ [54]. The four-fold difference between the Spanish study and our result could be due to their sample of 8 non-breeding adult birds, not tightened to a nest, and, most importantly, in an impoverished trophic context in Spain, that increased mortality and reduced breeding success in griffon vultures during several years [55], [56]. Indeed, breeding success in the Causses averaged 0.85 since 2007 (LPO, unpublished results) while it dropped below 0.3 in northern Spain in the same time period [55]. These vultures tracked in Spain may have been obliged to perform long-ranging movements in search for rare carcasses. It is thus difficult to conclude from the comparison of these two GPS studies that vultures in the Causses displayed small home ranges because of the particularly “good” trophic context of feeding stations, or if vultures in Spain displayed large home range because of the “bad” trophic context of lack of feeding sites.

All other studies were based on VHF radiotracking with only a few fixes per day and a low spatial precision [27], [57], [58]. In the Causses, a previous study on 22 griffon vultures based on radio-tracking found a home range area (fixed kernel method) in summer of 708 km^2^ ±184 km^2^, [27]. However, data were collected in 2003–2004 with a very small number of locations per individual (35±6 compared to 11 725±11 171 in our study). Moreover, the constant increase of the vulture populations in the Causses (118 breeding pairs in 2004 vs. 330 pairs in 2011), and of the number of light feeding stations (13 in 2004 at a maximal distance of 24.5 km from the colonies vs. 69 in 2011 at a maximal distance of 34.5 km), may partly explain this increase in home range size between 2004 and 2011. Further comparison of home range size from other study sites must be considered with caution since the topography, the vulture population size and the overall availability of food resources may play an important role in determining home range characteristics. For instance, the mean annual home range size of griffon vultures in Crete (692±299 km^2^, n = 7, using kernel method [58]), was smaller than ours (962±623 km^2^), but since Crete is a narrow island (maximum width of c. 50 km) the movements of vultures are certainly very restricted.

### Impact of Food Predictability

The temporal variability of food resources for vultures can be best understood by the seasonal variation in livestock mortality while spatial predictability was reflected by the type of habitat and feeding site used. In winter, the short daily distances observed in the Causses (29 km.day^−1^) were rather similar to those recorded in Israel (35 km.day^−1^) [59]. The short distances covered daily, and the low value of flatness of the UD (the ratio between the CA and home range areas, used as a proxy for the concentration of activity), indicated that the activity within the home range was very concentrated around the colonies. Winter was characterized by a low insulation (typical of Mediterranean climate [24]) that limited thermal soaring, albeit compensated by stronger winds that could create orographic ascending currents close to the relief and canyons. Moreover, short days reduced the time available for foraging. The resulting poor flight conditions may have forced vultures to reduce their foraging area, to concentrate their activity around specific places, especially at some feeding stations, as suggested by the increase in the density of feeding stations included in the home ranges in winter. However vultures may not be really disadvantaged by these poor flight conditions in their foraging activity because the high mortality in sheep herds (lambing period) in winter [22] provided abundant carcassses at feeding stations at short distance from the colonies [26]. The habitat selection analysis confirmed this hypothesis, since vultures in winter seemed to prefer spatially predictable resources (LFS and HFS) compared to other habitats where food was less predictable and almost nonexistent since most livestock herds are kept inside barns in that season.

In summer, in contrast, optimal flight conditions allowed vultures to spread their activity to a larger area. Vultures covered more distance per day (>100 km.day^−1^, larger than the figure (70 km.day^−1^) found in Israel [59]) and their home ranges contained more feeding stations, more diluted in the total area (decrease in the density of feeding stations). Although they still preferred predictable resources, the significant preference for open grassland compared to closed habitat suggested that vultures could also forage on randomly distributed resources (as confirmed by numerous reports of feeding events away from official feeding stations).

Spring foraging patterns were rather similar to summer ones, as expected since flight conditions were relatively similar during both seasons, although lambing period provided more dead livestock in spring than in summer. During chick rearing period, adult vultures need to feed their young frequently. Because carcasses were still plentiful in spring at every feeding station, breeding vultures may have favoured light feeding stations, not too far from colonies, where their chance to access food was probably higher than at a heavy feeding stations, characterized by intense competition [26]. According to the habitat selection analysis, vultures did not forage much on randomly distributed resources (open meadows) in spring, perhaps to increase efficiency in food finding, to maximize feeding rate of chicks, as observed in other central-place marine scavengers such as albatrosses [60].

Finally, in autumn, whereas the flatness of the Utilization Distribution and the density of feeding stations were similar to those observed in spring and summer, fewer feeding stations were encompassed within the home range, and the daily distance was smaller than in spring and summer. This could be explained by flight conditions that progressively deteriorate, and by the increase of the amount of food resources available at feeding stations. The habitat selection analysis showed no clear differences in the use of the different resources, perhaps because as breeding was finished, several related constraints were relaxed, enabling adults to forage more “freely”. As an example, once its chick fledged, one adult vulture undertook a long range foray to the Alps that lasted one week before coming back to its nest, as also observed in Israel or Spain [54], [59].

To sum up, when vultures are constrained by time and flight conditions (like in winter), or by low food availability and high reproductive investment (like in summer), they concentrate their search effort on habitats with highest likelihood to find carcasses, i.e. heavy feeding stations, even if competition to access food is harsh. On the other hand, when they are less constrained like in spring (good flight condition and abundant carcasses), they tend to favour light feeding stations, presumably because competition is reduced there [26].

### Age and Sex Variations in Ranging Behaviour

Contrary to expectations, we did not find differences in home ranges between adults and immatures. A previous study on the same population [27] reported that adults exhibited smaller home range size than immatures and juveniles, presumably due to their higher dominance rank during feeding events that gave them an easier access to food [26], forcing immatures to forage further away from the colonies (heavy feeding stations at that time were mostly located close to the breeding colonies). The absence of juvenile individuals in our study and the increase in the number of light feeding stations since the study of Gault [27] may explain the differences with our results. In 2004, 15 feeding stations were available to 118 breeding pairs (i.e. 7.9 pair per feeding station) while in 2010, the ratio was 69 feeding stations for 275 pairs, i.e. 3.9 pairs per feeding station. Thus the intraspecific competition has probably decreased between the two studies, presumably decreasing the need for young vultures to forage larger areas than adults. Yet a second hypothesis can be proposed. Between 2004 and 2010, while the breeding population almost tripled, the area covered by the colony did not expand much. This higher density of nests in the colony in 2010 may have forced breeding adults to extend their foraging range because they could not find enough food for all breeders at a close distance to their nest. To our knowledge, no comparison with other sites or with other vulture species is available, except for Californian condors (*Gymnogyps californianus*) where immatures forage over a larger area than adults [30].

Immatures were generally less selective regarding foraging habitat compared to adults. Immatures usually favoured habitats with predictable resources, but did not significantly prefer light over heavy feeding stations. Such results could be attributed to a lack of experience, but also to a lack of reproductive constraints. Anecdotal evidence provided by one immature vulture which visited all feeding stations within one month of its release suggests that lack of experience may not be a suitable explanation. The fact that immatures did not concentrate at feeding stations (especially the heavy ones) certainly enabled them to limit competition with adults during feeding events. However, any conclusion regarding a lack of age-related difference in home range characteristics would be premature given the small number of immatures tracked.

The absence of a sex differences on home range characteristics was expected, considering the very marginal sexual dimorphism and the equal investment of males and females in reproduction [28]. In vultures, a difference in foraging behavior was only found in species like Andean condors *Vultur gryphus* with large sexual dimorphism [61].

### Implication for Conservation

Feeding stations enabled vultures to adjust their foraging strategy according to their current energy needs and flight conditions. Outside of the winter months (when food resources are plentiful in feeding stations and when there is no resource available naturally in open habitats) and during periods with poor conditions for flying (eg rainy periods), feeding stations may supply vultures with easily accessible resources, demanding less distance to cover (thus less time or energy in flight) for foraging than habitats with unpredictable resources. Moreover, when energy needs were important (like during chick rearing), feeding stations provided a good support to easily find food, reducing distance travelled during foraging. Light feeding stations were always preferred over heavy ones, possibly because competition is lower [26] or because these feeding stations better simulate conditions that vultures would have experienced before this management [62]. The fact that vultures were also foraging over open habitats without feeding stations, searching randomly for resources, demonstrated that they had not lost their natural foraging ability and opportunistic behaviour. Indeed every year, vultures discovered new feeding sites. The best demonstration is certainly the discovery of the northernmost heavy feeding station (Fig. 1) that is a zoological park (“Les loups du Gévaudan”) dedicated to 50 wolves *Canis lupus*, for which almost a ton of meat is deposited every week in a meadow of 20-ha park. Vultures discovered the site for the first time in summer 2009 and fed there before wolves (the meat being deposited at midday while wolves prefer to feed at night). In summer 2011, more than 100 vultures visited the site every day (among which 10 vultures tracked by GPS in this study occurred regularly). Therefore the viability of the griffon vulture population reintroduced in the Causses is certainly not compromised by the habituation of vultures to feeding sites [15], [20], [63].

In conclusion, the problems related to (supplementary) feeding stations (concentration of individuals modifying foraging and breeding behaviour) highlighted for solitary and territorial vulture species [6], [18], [19] may not apply, or may apply to a lesser extent, in social vultures like *Gyps* vultures or Black vultures (*Coragyps atratus*). Promoting the development of feeding stations as in the Causses is certainly a good way of supporting vultures population for reintroduction programs, in areas of insufficient food or to facilitate recolonization of breeding sites, together with providing farmers with efficient and economical service of carcass elimination [23]. This solution could be an alternative to the complicated situation of vulture conservation in Europe, in a context of opposition between sanitary and environmental policies [56]. In accordance with a previous study based on ecological criteria [64], these results suggest that heavy feeding stations should be used initially when populations are small and competition is low and then replaced by light feeding stations, which limit competition through lower predictability [26]. However, since vultures seem to maintain their natural ability to forage on randomly distributed resources outside of feeding stations, this conservation tool may become inefficient when it is used as a mean to provide safe food and to prevent vultures from feeding on contaminated resources, such as in Asia [9]. In this case, efforts should be made on eliminating mortality causes and not only on providing healthy food resources, because vultures are likely to keep foraging on contaminated carcasses outside the feeding stations.
